# Mechanistic insights into atorvastatin-induced hepatotoxicity: molecular pathways, clinical relevance, and strategies for safer personalized therapy

**DOI:** 10.1007/s10238-026-02091-w

**Published:** 2026-03-05

**Authors:** Mohammed A. Abdel-Rasol, Wael M. El-Sayed

**Affiliations:** Department of Zoology, Faculty of Science, Ain Shams University, Abbassia, Cairo, 11566 Egypt

**Keywords:** Bile acid transporters, Genetic susceptibility, Inflammation, Mitochondrial dysfunction, Oxidative stress, Personalized medicine, SLCO1B1 polymorphisms

## Abstract

Atorvastatin, a chemically defined HMG-CoA reductase inhibitor, is widely prescribed for hyperlipidemia and cardiovascular disease prevention. However, it has been implicated in hepatotoxic effects ranging from transient transaminase elevations to rare but severe liver injury. This review critically examines the molecular and biochemical mechanisms underlying atorvastatin-induced hepatotoxicity, emphasizing translational relevance and human health risk assessment. A structured literature search (2000–2025) integrated evidence from clinical reports, experimental models, and pharmacogenomic studies. Key pathways analyzed included mitochondrial dysfunction, oxidative stress, bile acid dysregulation, and inflammatory signaling, with special attention to genetic polymorphisms (*SLCO1B1*,* CYP3A4*,* UGT1A1*) and drug–drug interactions. Atorvastatin-induced hepatotoxicity results from interconnected molecular events. Mitochondrial dysfunction impairs electron transport chain activity, causing ATP depletion and excessive ROS production. Oxidative stress drives lipid peroxidation, protein modification, and DNA injury, while inhibition of bile acid transporters (BSEP, NTCP, MRP2) promotes cholestatic damage. ROS and bile acid accumulation activate Kupffer cells and the NLRP3 inflammasome, amplifying inflammatory cascades (e.g., TNF-α, IL-1β). Pharmacogenomic variations in SLCO1B1, CYP3A4/5, and UGT1A1 modulate atorvastatin disposition and susceptibility, contributing to idiosyncratic injury. Drug–drug interactions further intensify hepatotoxic risk. Mechanistic insights support preventive strategies such as genotype-guided dosing, structured liver function monitoring, and adjunctive therapies targeting oxidative stress, mitochondrial stabilization, or bile acid homeostasis. Defining these mechanistic pathways provides a framework for integrating pharmacogenomic data and mechanistic biomarkers into clinical practice, enabling safer, more personalized statin therapy and improving risk stratification in drug-induced liver injury.

## Methodology

This narrative review employed a structured literature search to examine the molecular mechanisms, clinical implications, and management strategies associated with atorvastatin-induced hepatotoxicity, synthesizing findings from studies published between 2000 and 2025.

A comprehensive PubMed search was conducted using keywords including “atorvastatin,” “hepatotoxicity,” “oxidative stress,” “mitochondrial dysfunction,” “bile acid transporters,” “cytochrome P450,” “drug interactions,” “genetic polymorphisms,” “liver injury,” “NAFLD and/or MAFLD,” and “NASH and/or MASH.” Boolean operators (AND, OR) were applied to refine results, restricted to English-language, peer-reviewed articles encompassing systematic reviews, clinical trials, and relevant preclinical studies.

Studies were included if they addressed mechanisms such as oxidative stress, mitochondrial dysfunction, bile acid transport, inflammatory signaling, cytochrome P450 metabolism, or genetic polymorphisms (e.g., *SLCO1B1*,* CYP3A4*,* UGT1A1*) relevant to personalized therapy. Articles on clinical management approaches, including liver function monitoring, dose adjustments, and hepatoprotective strategies, were also considered. Both human studies and relevant preclinical models were included to provide a comprehensive perspective. Limitations include heterogeneity in study designs, populations, and outcome measures, which may affect generalizability and preclude definitive causal conclusions.

## Introduction

Atorvastatin, a widely used statin, lowers LDL cholesterol by inhibiting 3-hydroxy-3-methylglutaryl coenzyme A (HMG-CoA) reductase, a key enzyme in hepatic cholesterol synthesis. This reduces mevalonate production, aiding in the management of hyperlipidemia and prevention of atherosclerotic cardiovascular diseases (CVDs), including myocardial infarction, stroke, and peripheral artery disease [1]. It is prescribed for both primary and secondary prevention of cardiovascular events and has consistently reduced cardiovascular morbidity and mortality worldwide [2].

Despite its benefits, atorvastatin can cause hepatic side effects. Elevated alanine aminotransferase (ALT) and aspartate aminotransferase (AST) levels serve as markers of hepatocellular stress, with ALT being more liver-specific. Most abnormalities are mild, reversible, and asymptomatic, but rare cases of severe liver injury, including failure, have been reported [3].

The mechanisms of hepatotoxicity remain incompletely understood but involve genetic predisposition, oxidative stress, mitochondrial dysfunction, and disrupted lipid metabolism. Metabolism by CYP3A4 may generate reactive intermediates contributing to liver injury [4]. These effects vary by individual genetics, drug interactions, and environmental exposures. Mitochondrial impairment, in particular, may disrupt energy balance and increase oxidative damage [5]. Current evidence recognizes atorvastatin’s hepatotoxic potential but lacks an integrated view of its molecular mechanisms and clinical relevance. Risk stratification remains limited. Personalized medicine, including pharmacogenomic approaches, offers a way to tailor therapy and reduce adverse outcomes [6].

While clinically significant hepatotoxicity is rare (0.1–2%), transient enzyme elevations are more common, especially in the first year [7]. Monitoring liver function is essential, particularly in high-risk patients or those taking other hepatotoxic drugs, as concerns over liver safety may affect adherence and compromise cardioprotective benefits. Consistent with product labeling in the US and other regions, baseline liver function tests are recommended prior to therapy, with subsequent testing guided by symptoms. Although this review focuses on hepatotoxicity, statins can also rarely cause rhabdomyolysis and secondary kidney injury.

This review synthesizes current evidence on the molecular and clinical mechanisms of atorvastatin-induced hepatotoxicity, emphasizing genetic susceptibility, transporter interactions, and mitochondrial dysfunction. It also explores clinical implications and management strategies, with a focus on personalized approaches such as pharmacogenomic testing and risk stratification to enhance therapeutic safety.

## Pathophysiology and mechanisms of hepatotoxicity

### Mitochondrial dysfunction

Atorvastatin is primarily metabolized in the liver, where it inhibits HMG-CoA reductase to lower cholesterol [8]. Beyond lipid-lowering, it can induce mitochondrial dysfunction—a key mechanism of hepatotoxicity. Mitochondria maintain energy production and metabolic balance; their impairment leads to defective β-oxidation, oxidative stress, apoptosis, and inflammation [9]. Mitochondria are the primary source of cellular energy in hepatocytes, generating ATP through oxidative phosphorylation, which is required to sustain normal liver function. Impaired mitochondrial energy production disrupts cellular bioenergetics, compromising ATP supply and destabilizing metabolic homeostasis. Such disturbance of mitochondrial energy balance contributes to hepatocyte stress, amplifies oxidative damage, and sensitizes cells to inflammatory and apoptotic signaling in liver injury [10]. Metabolic disorders affecting mitochondrial function may activate hepatic stellate cells (HSCs), promoting fibrogenic pathways and extracellular matrix accumulation, thereby accelerating fibrosis progression and potentially leading to cirrhosis and liver failure if unresolved [10].

#### Impairment of mitochondrial β-Oxidation

**Fig. 1:**
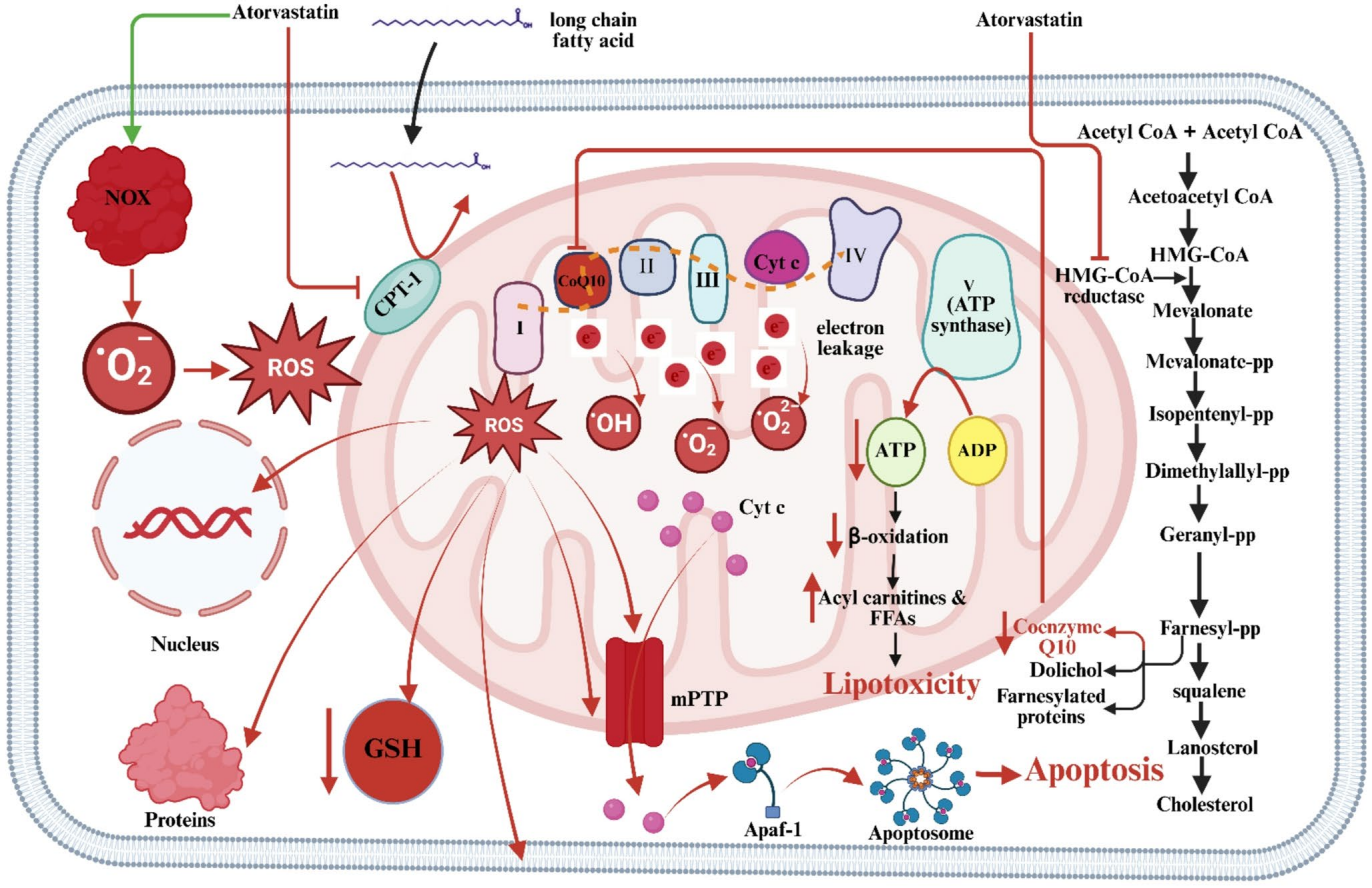
Mechanisms of Atorvastatin-Induced Mitochondrial Dysfunction, Oxidative Stress, and Hepatotoxicity. CoQ10: Coenzyme Q10, CPT-1: Carnitine Palmitoyltransferase-1, FFA: Free Fatty Acids, GSH: Glutathione, mPTP: Mitochondrial Permeability Transition Pore, NOX: NADPH Oxidase, ROS: Reactive Oxygen Species. **→**: Activation, **┬**: Inhibition, **↓**: Decrease, **↑**: Increase

Atorvastatin disrupts β-oxidation by reducing CoQ10 via mevalonate pathway inhibition [11], decreasing mitochondrial respiratory activity by approximately 30–40% in experimental models, impairing the electron transport chain and lowering ATP production in a dose-dependent manner. Incomplete fatty acid oxidation results in toxic lipid intermediates (e.g., acyl-carnitines, FFAs), promoting lipotoxicity and mitochondrial damage [12]. It may also inhibit carnitine palmitoyltransferase-1, impeding fatty acid transport into mitochondria [13], thus worsening FFA accumulation and hepatocellular injury (Fig. 1).

#### Oxidative stress and reactive oxygen species (ROS) generation

CoQ10 depletion also increases ROS generation due to ETC inefficiency [14], resulting in up to a 2-fold rise in ROS levels and subsequent oxidative injury. ROS damage lipids, proteins, and DNA, compromising cell integrity. Atorvastatin further depletes antioxidants like glutathione (GSH), intensifying oxidative stress [15]. ROS generation is amplified by NADPH oxidase activity, promoting H₂O₂ and •OH production [9]. This leads to mitochondrial membrane disruption, mitochondrial permeability transition pore (mPTP) opening, cytochrome c release, and intrinsic apoptosis [11].

#### Inflammatory pathways and liver injury

**Fig. 2:**
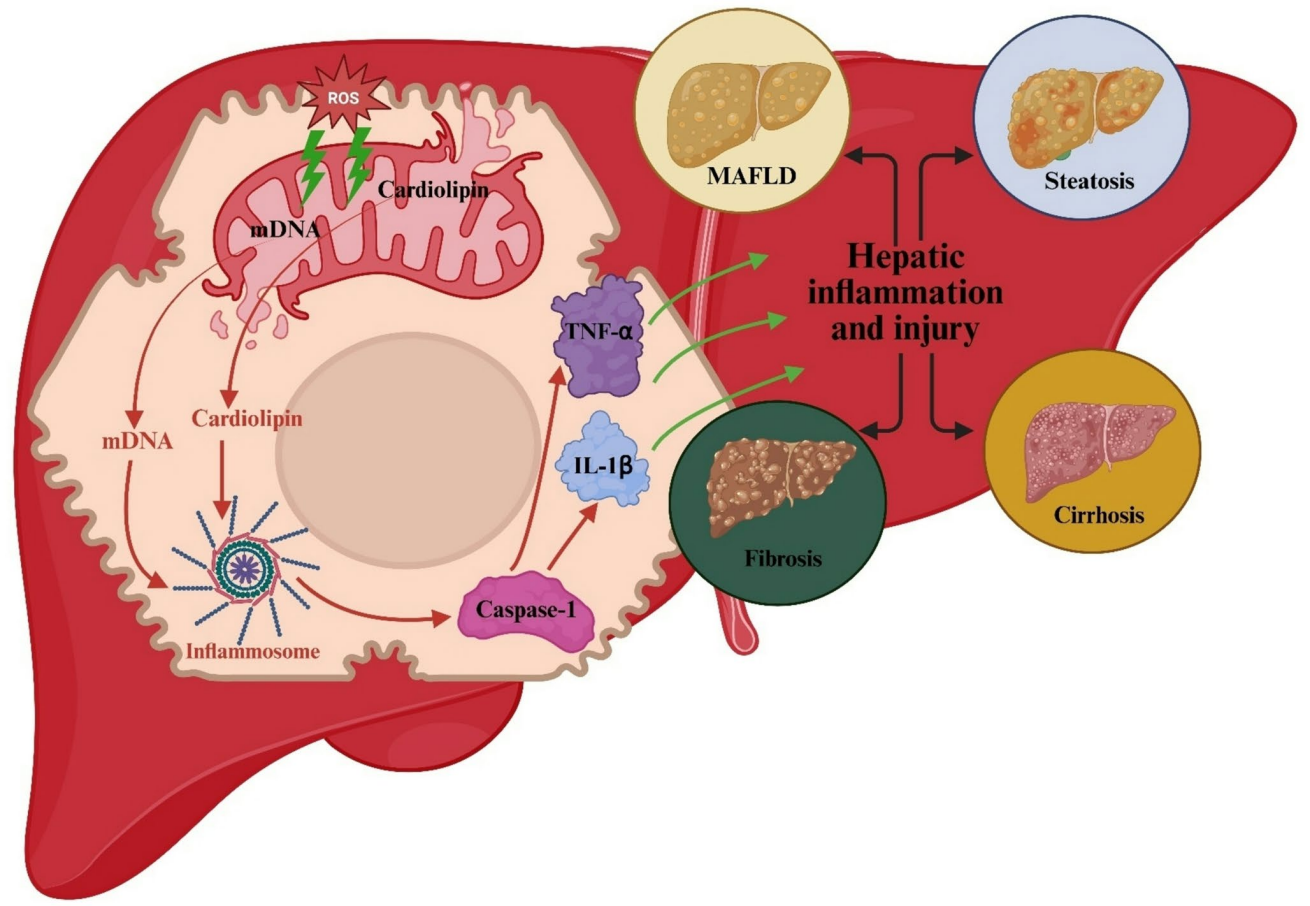
Inflammatory Pathways Activated by Atorvastatin-Induced Mitochondrial Damage Leading to Liver Injury and Disease Progression. IL-1β: Interleukin-1 Beta, mDNA: mitochondrial DNA, MAFLD: Metabolic Dysfunction–Associated Fatty Liver Disease, ROS: Reactive Oxygen Species, TNF-α: Tumor Necrosis Factor Alpha

Mitochondrial damage releases damage-associated molecular patterns (DAMPs)—such as mitochondrial DNA and cardiolipin—that trigger inflammasomes and activate caspase-1, interleukin-1 beta (IL-1β) and tumor necrosis factor-alpha (TNF-α) [9, 12]. These cytokines perpetuate liver inflammation and injury, contributing to MAFLD, steatosis, and MASH [16]. MAFLD provides a useful framework for understanding liver injury by highlighting the combined impact of metabolic disturbances and coexisting conditions, such as alcohol-related liver disease (ARLD) and viral hepatitis. This dual etiology underscores the complexity of hepatotoxicity in MAFLD. Liver injury in MAFLD arises from lipid metabolism disruptions, leading to fat accumulation, inflammation, and mitochondrial dysfunction, which exacerbate hepatocyte damage and fibrosis. Coexisting conditions, such as autoimmune hepatitis (AIH) or viral hepatitis, can further complicate treatment and accelerate liver damage [17, 18]. In predisposed individuals, inflammation may advance to fibrosis or cirrhosis [19]. Thus, mitochondrial dysfunction and inflammation jointly drive acute and chronic liver pathology (Fig. 2). While mild oxidative and inflammatory responses may correlate with atorvastatin dose, clinically significant hepatotoxicity remains largely idiosyncratic, particularly in genetically susceptible individuals.

#### Genetic and environmental modifiers

Genetic polymorphisms, such as in SOD2, impair ROS detoxification and increase hepatotoxic risk [20]. CYP3A4 inhibitors (e.g., macrolides) raise atorvastatin levels, enhancing mitochondrial exposure and toxicity [4]. Polymorphisms in SLCO1B1 that reduce OATP1B1 function limit hepatic uptake of atorvastatin, leading to higher plasma concentrations and prolonged systemic exposure. This increases hepatocyte susceptibility to oxidative and mitochondrial injury, especially when combined with CYP3A4 inhibition or environmental risk factors. These gene–environment interactions heighten susceptibility to liver injury.

Atorvastatin-induced hepatotoxicity arises from impaired β-oxidation, oxidative stress, inflammation, and individual susceptibility. Recognizing these mechanisms enables targeted strategies like genetic screening, antioxidant supplementation, and drug interaction avoidance—helping mitigate risk while maintaining cardiovascular protection.

### Cytochrome P450 enzyme interaction

#### Metabolism of Atorvastatin

Atorvastatin is metabolized in the liver by CYP3A4. Inhibition of CYP3A4—by drugs such as macrolides, azole antifungals, protease inhibitors, or grapefruit juice—raises atorvastatin levels, heightening the risk of hepatotoxicity and myopathy [4]. Elevated concentrations impair mitochondrial function by depleting CoQ10, reducing ATP production, and increasing ROS generation (e.g., H₂O₂, O₂•⁻) [15]. ROS trigger mPTP opening, cytochrome c release, and intrinsic apoptosis, leading to hepatocyte death [21].

ROS also activate inflammasomes, promoting IL-1β and TNF-α release and fostering hepatic inflammation [10], which can contribute to chronic liver diseases such as MAFLD and MASH. Excess atorvastatin may also affect muscle, increasing risk of myopathy and rhabdomyolysis through similar oxidative and mitochondrial pathways [22].

In summary, CYP3A4 inhibition significantly elevates atorvastatin levels, leading to mitochondrial dysfunction, inflammation, and systemic toxicity. Recognizing drug and dietary interactions is crucial to prevent adverse outcomes.

### Bile acid transporter Inhibition

Atorvastatin also impairs hepatic bile acid transport, leading to intracellular accumulation and cholestasis [23]. This exacerbates hepatotoxicity via bile acid-induced membrane damage, inflammation, and apoptosis.

#### Hepatic bile acid transporters affected by Atorvastatin

##### Bile salt export pump (BSEP, ABCB11)

BSEP, critical for canalicular bile acid excretion, is downregulated by atorvastatin [24–26], with experimental studies showing 40–60% reduction in expression, leading to bile retention [27]. FXR activation initially suppresses bile acid synthesis but ultimately inhibits BSEP itself, worsening bile overload [28]. Retained bile acids activate MAPK pathways (JNK, p38), inducing cytokines (TNF-α, IL-1β, IL-6) that drive inflammation and liver injury [29]. Prolonged cholestasis leads to mitochondrial dysfunction and caspase-mediated apoptosis, increasing risk of fibrosis and cirrhosis [14] (Table 1).

**Table 1:** Effects of Atorvastatin on hepatic bile acid transporters and liver dysfunction

Transporter	Location	Function	Effect of Atorvastatin	Resulting Liver Dysfunction	Associated Signaling Pathways
Bile Salt Export Pump (BSEP) (ABCB11)	Canalicular membrane of hepatocytes	Exports conjugated bile acids (taurocholate, glycocholate) from hepatocytes into bile	Inhibits BSEP expression and activity, leading to bile acid accumulation and cholestasis	Bile acid-induced cytotoxicity, mitochondrial dysfunction, cholestasis, liver fibrosis, cirrhosis	FXR, MAPK (JNK, p38), NF-kB, caspase-dependent apoptosis
Multidrug Resistance-Associated Proteins (MRP2 and MRP3)	Canalicular membrane (MRP2), Basolateral membrane (MRP3)	Exports conjugated bile acids and bilirubin into bile (MRP2), exports bile acids into the bloodstream (MRP3)	Downregulates MRP2, upregulates MRP3, contributing to bile acid accumulation and systemic toxicity	Bile acid overload, systemic bile acid toxicity, liver inflammation, fibrosis, cirrhosis	FXR, Nrf2, NF-kB, JNK, MAPK, systemic toxicity
Organic Anion Transporting Polypeptides (OATP1B1/1B3)	Sinusoidal membrane of hepatocytes	Bile acid uptake from bloodstream into hepatocytes (OATP1B1), exports bile acids into bloodstream (OATP1B3)	Inhibits OATP1B1, leading to bile acid accumulation in hepatocytes and systemic toxicity	Bile acid toxicity, liver dysfunction, inflammation, fibrosis, cirrhosis	FXR, NF-kB, JNK, oxidative stress, MAPK
Sodium-Taurocholate Cotransporting Polypeptide (NTCP) (SLC10A1)	Basolateral (sinusoidal) membrane of hepatocytes	Uptakes conjugated bile acids (taurocholate) from bloodstream into hepatocytes	Inhibits NTCP expression and activity, leading to impaired bile acid uptake and cholestasis	Bile acid accumulation, liver fibrosis, inflammation, cirrhosis	FXR, NF-kB, JNK, oxidative stress

BSEP: Bile Salt Export Pump, MRP2: Multidrug Resistance-Associated Protein 2, MRP3: Multidrug Resistance-Associated Protein 3, OATP1B1: Organic Anion Transporting Polypeptide 1B1, OATP1B3: Organic Anion Transporting Polypeptide 1B3, NTCP: Sodium-Taurocholate Cotransporting Polypeptide, FXR: Farnesoid X Receptor, MAPK: Mitogen-Activated Protein Kinase, JNK: c-Jun N-terminal Kinase, NF-κB: Nuclear Factor Kappa B, PPARα: Peroxisome Proliferator-Activated Receptor Alpha

##### Multidrug Resistance-Associated proteins (MRP2 and MRP3)

MRP2 exports conjugated bile acids and bilirubin, while MRP3 compensates during impaired bile flow [30]. Atorvastatin suppresses MRP2 via oxidative stress and Nrf2 inhibition, compounded by FXR signaling [15]. MRP3 upregulation helps mitigate hepatocyte bile acid overload but elevates systemic bile acid levels, which may damage kidneys, intestines, and trigger systemic inflammation [31] (Table 1).

##### Organic anion transporting polypeptides (OATP1B1 and OATP1B3)

These sinusoidal transporters mediate hepatic bile acid uptake. Atorvastatin inhibits OATP1B1, causing intracellular bile acid buildup [4]. Importantly, SLCO1B1 polymorphisms that reduce OATP1B1 activity further decrease hepatic atorvastatin uptake, amplifying systemic exposure and increasing the risk of hepatotoxicity. In compensation, OATP1B3 is upregulated via FXR signaling but contributes to systemic bile acid burden and inflammation through NF-κB, JNK, MAPK, and PPARα pathways [32] (Table 1).

##### Sodium-Taurocholate cotransporting polypeptide (NTCP) (SLC10A1)

NTCP facilitates hepatic uptake of conjugated bile acids. Atorvastatin suppresses NTCP expression and activity, leading to increased systemic bile acid levels and aggravated cholestasis [33]. FXR signaling and atorvastatin-induced oxidative stress further downregulate NTCP via NF-κB and JNK pathways [34].

Together, these disruptions to bile acid transporters (BSEP, MRP2/3, OATP1B1/1B3, NTCP) result in hepatocyte bile acid overload, oxidative stress, and systemic toxicity. Pathways including FXR, MAPK, NF-κB, JNK, and PPARα are activated, promoting inflammation, fibrosis, and potentially cirrhosis. Monitoring liver function and transporter interactions is essential in at-risk patients (Table 1).

#### Mechanisms of bile acid toxicity

Inhibition of bile acid transporters—including BSEP, NTCP, and MRP2—by atorvastatin and its metabolites results in marked intrahepatic accumulation of hydrophobic bile acids (e.g., CDCA, DCA, LCA). These bile acids act as detergents, destabilizing membranes and inducing a 2–3-fold increase in ROS production and lipid peroxidation reported in experimental models [35]. This oxidative burden, alongside pro-inflammatory signaling, drives hepatocellular necrosis and apoptosis rather than being purely adaptive.

**Fig. 3:**
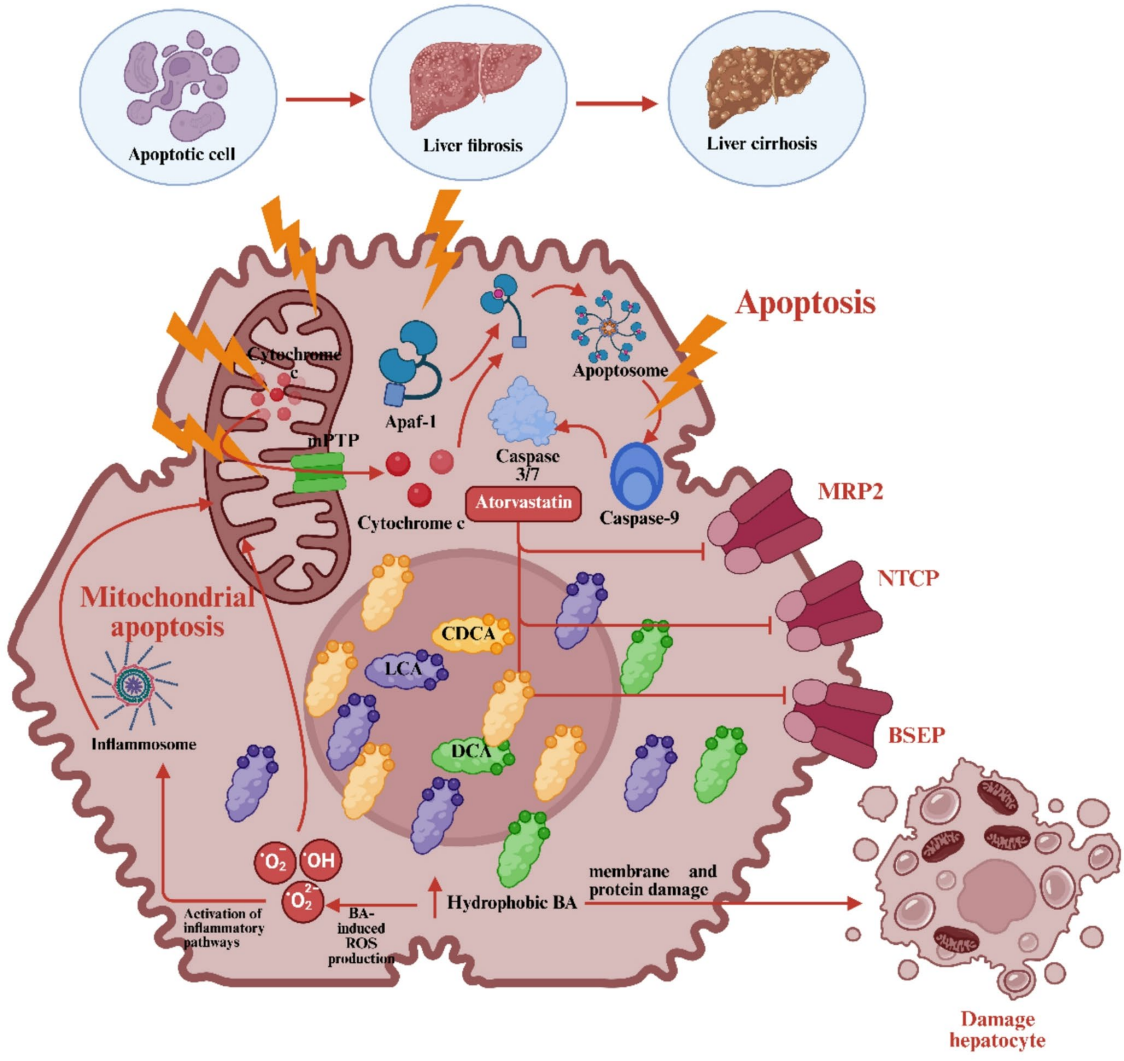
Mechanisms of Atorvastatin-Induced Bile Acid Toxicity in Hepatocytes. BA: Bile Acid, BSEP: Bile Salt Export Pump, NTCP: Sodium Taurocholate Cotransporting Polypeptide, MRP2: Multidrug Resistance–associated Protein 2, CDCA: Chenodeoxycholic Acid, DCA: Deoxycholic Acid, LCA: Lithocholic Acid, mPTP: Mitochondrial Permeability Transition Pore. **→**: Activation, ┬: Inhibition, **↑**: Increase

As damage accumulates, hepatic detoxification capacity declines. Mitochondrial dysfunction and apoptotic signaling escalate hepatocyte loss, contributing to fibrosis and cirrhosis [36]. Figure 3 illustrates these cellular events.

##### Direct cellular damage by hydrophobic bile acids

Hydrophobic bile acids like CDCA and DCA destabilize mitochondrial membranes and inhibit ETC complex I and III activity by approximately 40–60% in vitro, reducing ATP production and causing energy depletion [37]. These bile acids also promote mPTP opening, cytochrome c release, and activation of caspase-3 and − 9 [29].

ETC disruption increases electron leakage and ROS generation. Superoxide production can increase 2–5 fold, which is subsequently converted to hydrogen peroxide (H₂O₂) and hydroxyl radicals (•OH), damaging mitochondrial DNA and lipids [38].

In parallel, bile acids activate death receptors like FAS and TRAIL-R2, initiating FADD-mediated caspase-8 activation and Bid cleavage, further amplifying mitochondrial permeabilization [39, 40]. Additionally, bile acids provoke ER stress and the unfolded protein response (UPR), sensitizing hepatocytes to apoptosis.

The convergence of mitochondrial dysfunction, oxidative stress, ER stress, and apoptosis contributes to cholestasis, fibrosis, and cirrhosis [41]. Although ROS production correlates with bile acid concentration, clinically significant injury remains largely idiosyncratic and amplified in genetically susceptible individuals (e.g., SLCO1B1 variants).

##### Oxidative stress and inflammation

CYP3A4 and CYP2E1 intensify bile acid toxicity by converting CDCA and LCA into more toxic derivatives such as 3-oxo-CDCA [42]. LCA is particularly harmful due to its ability to form DNA adducts and decrease PARP-1 activity by ~ 40%, impairing DNA repair [43]. These biotransformations deplete NADPH and GSH, weakening antioxidant defenses and elevating ROS levels.

Persistent bile acid accumulation activates Kupffer cells through TLR4, especially in the presence of LPS, and bile acid crystals activate the NLRP3 inflammasome, producing TNF-α, IL-1β, and IL-6 [44]. Membrane-bound O-acyltransferase domain-containing 7 (MBOAT7) has recently emerged as a novel regulator of TLR signaling, playing a pivotal role in modulating inflammation. MBOAT7 influences TLR function through its involvement in lipid metabolism. It transfers acyl groups to membrane lipids, affecting membrane fluidity and the signaling properties of TLRs. This modulation can alter TLR sensitivity to external stimuli, thereby controlling the strength and duration of the inflammatory response [45].

Inflammation is further amplified by NETosis, which promotes tissue injury and fibrogenesis [46]. This inflammatory amplification provides a mechanistic basis for the dose-idiosyncratic duality of atorvastatin-induced cholestatic injury.

##### Impaired detoxification

Phase II detoxification pathways—particularly sulfation and glucuronidation—are quantitatively downregulated during bile acid overload. SULT2A1 activity falls by ~ 50%, resulting in unsulfated bile acid accumulation [47]. UDPGT1A1 and UDPGT2B4 activity is also suppressed [48]. Although FXR activation partially compensates, ROS further inactivates UDPGTs post-translationally, aggravating bile acid buildup [21].

Basolateral exporters (MRP3, MRP4) become saturated, and MRP2 inhibition by atorvastatin exacerbates bilirubin retention, contributing to hyperbilirubinemia [49] (Table 2).

**Table 2:** Impaired detoxification mechanisms in Atorvastatin-Induced liver injury

Detoxification Process	Normal Function	Effect of Atorvastatin	Resulting Liver Dysfunction	Associated Pathways
Sulfation by SULT2A1	Sulfates bile acids (e.g., LCA) to less toxic forms (e.g., lithocholate sulfate)	Suppressed SULT2A1 expression leads to unsulfated toxic bile acids	Bile acid overload, hydrophobic bile acid accumulation, hepatotoxicity	FXR activation, mitochondrial dysfunction, apoptosis activation
Glucuronidation by UGT1A1 and UGT2B4	Detoxifies bilirubin and bile acids (UGT1A1); detoxifies CDCA and DCA (UGT2B4)	Impaired glucuronidation due to inhibited UGT1A1 and UGT2B4 expression	Increased toxicity from bile acid accumulation, impaired detoxification	FXR repression of UGT1A1, oxidative stress
Oxidative Stress and ROS Activation	Activates detoxification enzymes, maintains liver homeostasis	ROS-mediated inactivation of UGT enzymes, enhancing bile acid toxicity	Increased ROS production, mitochondrial dysfunction, DNA damage	NF-kB, JNK activation, mitochondrial stress, apoptosis
Bile Acid Export via MRP3/4	Exports bile acids from hepatocytes to prevent accumulation in the liver	Overwhelmed bile acid export systems, leading to systemic bile acid toxicity	Hyperbilirubinemia, jaundice, exacerbated liver injury	Systemic bile acid spillover, jaundice, inflammation

SULT2A1: Sulfotransferase Family 2 A Member 1, UGT1A1: UDP-Glucuronosyltransferase 1A1, UGT2B4: UDP-Glucuronosyltransferase 2B4, FXR: Farnesoid X Receptor, MRP3: Multidrug Resistance-Associated Protein 3, MRP4: Multidrug Resistance-Associated Protein 4, ROS: Reactive Oxygen Species, NF-κB: Nuclear Factor Kappa B, JNK: c-Jun N-terminal Kinase

### Apoptosis pathways

Caspase-dependent apoptosis via MOMP is central to atorvastatin-induced hepatotoxicity. Cytochrome c release initiates apoptosome formation with Apaf-1 and procaspase-9, activating caspase-9 and downstream executioner caspases like caspase-3 and − 7, culminating in dose-responsive hepatocyte apoptosis but often clinically idiosyncratic presentation [50].

Atorvastatin increases pro-apoptotic Bcl-2 proteins (Bax, Bak, Bid) and suppresses anti-apoptotic ones (Bcl-2, Bcl-xL), favoring MOMP [51]. Mitochondrial depolarization and cytochrome c release have been shown to increase by 2–4 fold in hepatocyte models, linking molecular events to clinical outcomes.

Elevated ROS activates ASK1 and JNK, amplifying apoptotic signaling [52]. ER stress contributes via IRE1α-mediated JNK activation, enhancing FAS ligand transcription and Bax translocation, while JNK inhibition of Bcl-2 reinforces apoptosis [53].

**Fig. 4:**
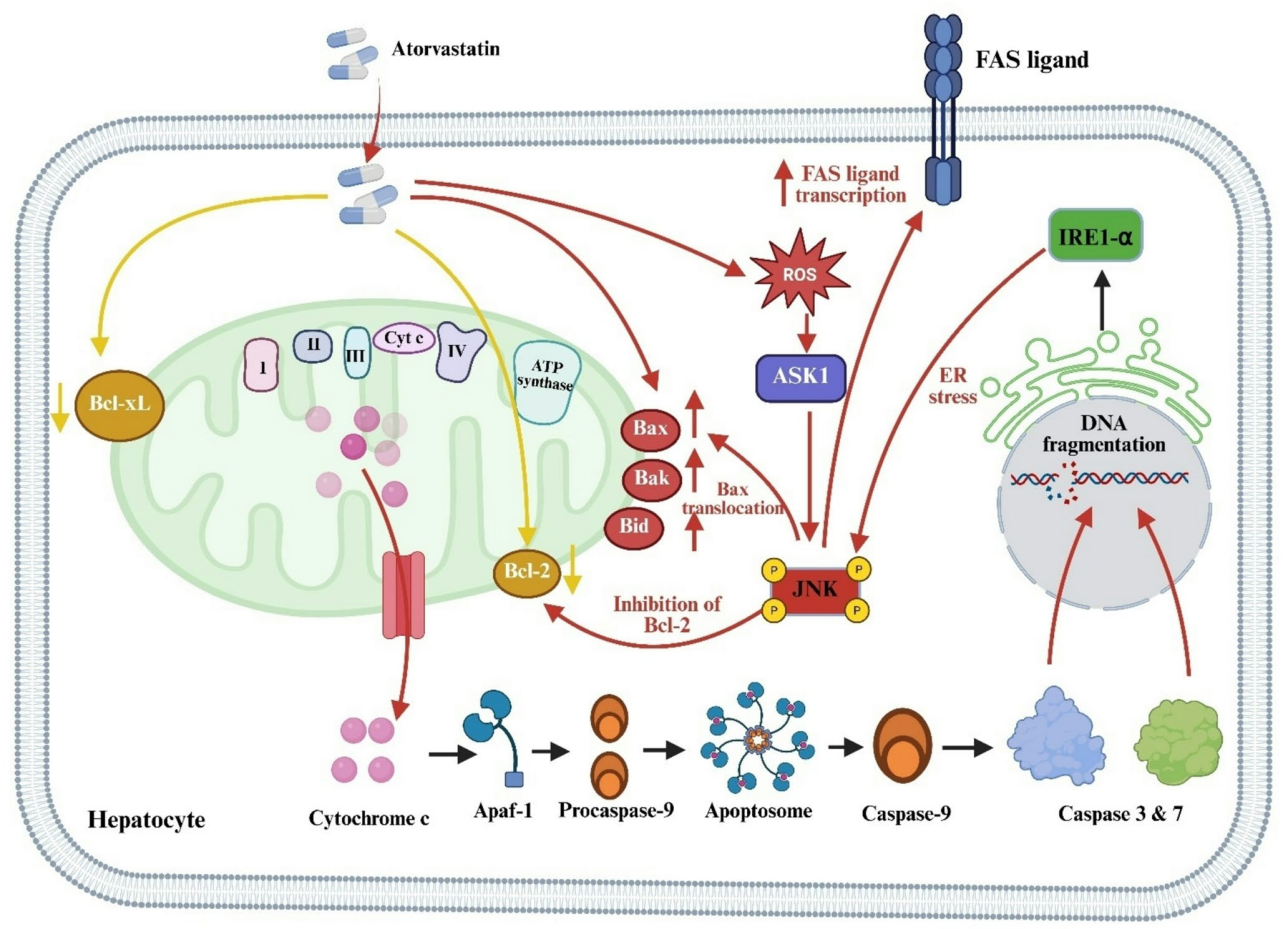
Caspase-Dependent Apoptosis Pathways in Atorvastatin-Induced Hepatotoxicity via Mitochondrial Dysfunction and ER Stress. Apaf-1: Apoptotic Protease Activating Factor 1, ASK1: Apoptosis Signal-Regulating Kinase 1, Bax: Bcl-2-associated X protein, Bak: Bcl-2 homologous antagonist/killer, Bid: BH3 Interacting Domain Death Agonist, Bcl-2: B-cell lymphoma 2, Bcl-xL: B-cell lymphoma-extra large, Caspase: Cysteine-aspartic protease, ER: Endoplasmic Reticulum, FAS: First apoptosis signal, JNK: c-Jun N-terminal kinase, ROS: Reactive Oxygen Species. **→**: Activation, **┬**: Inhibition, **↓**: Decrease, **↑**: Increase

This mechanistic integration provides quantitative context and clearly distinguishes pathway activation from interindividual susceptibility (Fig. 4).

**Fig. 5:**
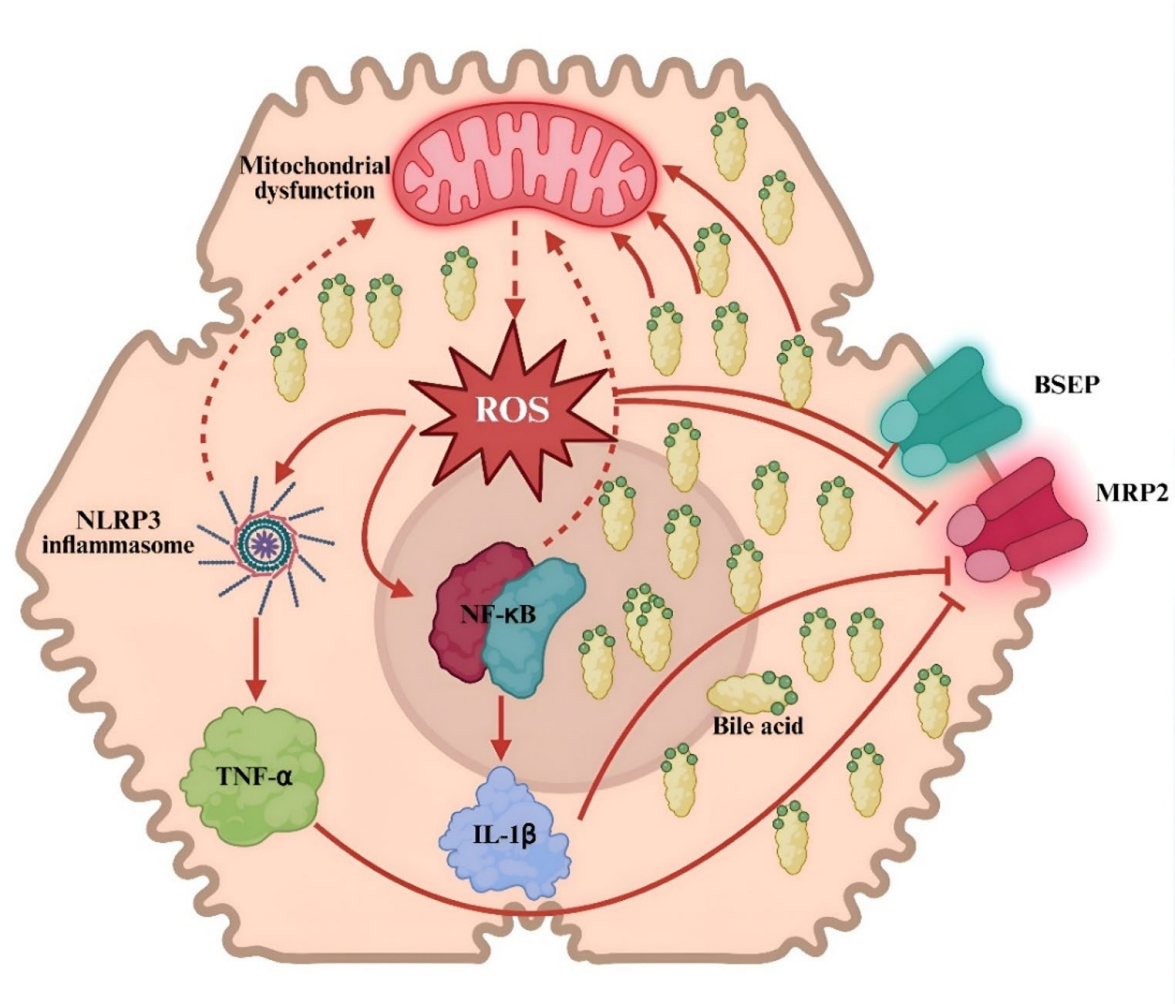
Integrated mechanistic crosstalk in atorvastatin-induced hepatotoxicity. Atorvastatin triggers mitochondrial dysfunction, leading to excess ROS generation, which in turn activates NF-κB and NLRP3 inflammasome signaling. This results in the release of pro-inflammatory cytokines (TNF-α, IL-1β) and suppression of bile acid transporters (BSEP, MRP2), promoting bile acid accumulation and further mitochondrial stress. Feedback loops amplify injury progression. Solid arrows indicate direct effects, dashed arrows indicate feedback loops, and blunt ends denote inhibition

Figure 5 presents an integrated crosstalk model linking mitochondrial dysfunction, ROS generation, inflammatory signalling, and bile-acid transporter disruption. The diagram emphasizes feedback loops whereby ROS and cytokines amplify transporter inhibition and mitochondrial injury, consistent with experimental and clinical evidence.

### Genetic susceptibility

Genetic variants modulate atorvastatin pharmacokinetics, influencing hepatotoxicity risk. Polymorphisms in SLCO1B1, CYP3A4/5, and UDPGT1A1 alter drug metabolism, detoxification, and transport, affecting individual susceptibility.

#### SLCO1B1 (OATP1B1) polymorphisms

The rs4149056 (T > C) variant reduces OATP1B1 function, impairing hepatic atorvastatin uptake and increasing plasma levels [54]. CC genotype carriers show 2–4× higher AUC, elevating the risk of hepatotoxicity via enhanced inhibition of bile acid transporters and oxidative stress [20, 55]. Though often associated with myopathy, this polymorphism’s hepatic relevance is increasingly recognized. Elevated atorvastatin/metabolite levels intensify ROS generation and mitochondrial injury, overwhelming cellular defenses and triggering inflammatory cascades. An overview of major genetic variants influencing atorvastatin disposition—including SLCO1B1, CYP3A4/5, and UGT1A1 polymorphisms—is summarized in Table 3. Genotype-guided dosing is advised—high doses (> 80 mg/day) are generally avoided in CC carriers [56]. Comparatively, hydrophilic statins like rosuvastatin exhibit lower dependence on OATP1B1, partially explaining their reduced hepatotoxicity in similar genetic backgrounds.

**Table 3:** Genetic susceptibility to Atorvastatin-Induced hepatotoxicity

Polymorphism	Gene/Enzyme	Variant and Effect	Population Prevalence	Clinical Implications
SLCO1B1 (OATP1B1) Polymorphisms (rs4149056)	SLCO1B1 (OATP1B1) transporter	rs4149056 (T > C) leads to Val174Ala substitution, reduced OATP1B1 function, higher atorvastatin plasma levels, increased hepatotoxicity risk.	Common in individuals with higher risk of hepatotoxicity, especially in those with the CC genotype.	Higher atorvastatin concentrations increase hepatotoxicity and myopathy risk; lower dosing recommended for CC genotype carriers.
CYP3A4 Polymorphisms (^*^CYP3A4 22)	CYP3A4 enzyme	rs35599367 (^*^CYP3A4 22) reduces CYP3A4 activity, leading to slower metabolism of atorvastatin and higher plasma concentrations.	5–10% of European populations carry the variant, leading to reduced atorvastatin clearance.	Reduced atorvastatin clearance increases liver injury risk, especially in individuals with the ^*^CYP3A4 22 variant.
CYP3A5 Polymorphisms (^*^CYP3A5 3)	CYP3A5 enzyme	rs776746 (^*^CYP3A5 3) causes non-functional CYP3A5 enzyme in poor metabolizers, resulting in elevated atorvastatin plasma concentrations.	Prevalent in 80–90% of Europeans as poor metabolizers, leading to elevated atorvastatin concentrations.	Poor metabolizers experience higher atorvastatin concentrations, increasing the risk of hepatotoxicity and mitochondrial dysfunction.
UGT1A1 Polymorphisms (UGT1A1 28 and UGT1A1 6)	UGT1A1 enzyme	UGT1A1 28 reduces glucuronidation capacity, impairing bilirubin and atorvastatin metabolite detoxification, leading to Gilbert’s syndrome and hepatotoxicity.	UGT1A1 28 is common in populations, particularly in those with Gilbert’s syndrome; UGT1A1 6 is prevalent in Asian populations.	Impaired detoxification of atorvastatin metabolites increases hepatotoxicity risk, particularly in individuals with Gilbert’s syndrome.

SLCO1B1: Solute Carrier Organic Anion Transporter 1B1, OATP1B1: Organic Anion Transporting Polypeptide 1B1, CYP3A4: Cytochrome P450 3A4, CYP3A5: Cytochrome P450 3A5, UGT1A1: UDP-Glucuronosyltransferase 1A1

#### CYP3A4/5 polymorphisms

CYP3A4*22 and CYP3A5*3 variants reduce enzyme activity, slowing atorvastatin metabolism [54]. CYP3A4*22 decreases hepatic clearance by approximately 20–30%, raising hepatotoxicity risk via increased systemic exposure [57]. CYP3A53 creates non-functional enzymes, classifying most European carriers as poor metabolizers (PMs) [58]. UMs may accumulate toxic intermediates, particularly when detox pathways are compromised [59]. Pharmacogenetic screening enables tailored therapy: PMs require lower doses, and UMs require close monitoring. Population prevalence varies—e.g., CYP3A53 is common in Europeans, while functional alleles are more prevalent in Africans—reinforcing its relevance for personalized therapy [60] (Table 3).

#### UDPGT1A1 polymorphisms

UDPGT1A1*28, involving TA-repeat expansion, reduces glucuronidation, impairing bilirubin and atorvastatin lactone clearance. This increases hepatotoxicity risk, particularly in those with Gilbert’s syndrome [61]. Affected individuals may show mixed hepatocellular/cholestatic patterns and elevated transaminases [3]. In Asians, UDPGT1A1*6 (Gly71Arg) is more common and similarly reduces detox capacity [62]. These polymorphisms shift the hepatotoxicity pattern toward idiosyncratic injury rather than strict dose dependence. Genotyping supports safer, more personalized atorvastatin therapy (Table 3).

### Lysosomal dysfunction and impaired autophagy

Lysosomal dysfunction and impaired autophagy are central to atorvastatin-induced hepatotoxicity [63]. Atorvastatin disrupts autophagy by inhibiting Rab GTPase geranylgeranylation, which is critical for lysosomal trafficking and acidification, thereby impairing degradation of damaged mitochondria [9]. Accumulation of dysfunctional mitochondria increases ROS levels up to 3–5 fold, worsening oxidative stress and mitochondrial injury. Elevated LC3-II and p62/SQSTM1 indicate impaired autophagic flux [64]. Impaired autophagy also hinders lipophagy, causing lipid droplet accumulation and hepatic steatosis [65]. This dysfunction favors persistent inflammatory signaling, compounding hepatocyte injury [66, 67]. Therapeutic targeting of autophagy may help protect genetically or metabolically vulnerable individuals.

### Inflammasome activation (NLRP3-Driven)

NLRP3 inflammasome activation is a key mechanism of atorvastatin-induced hepatotoxicity. Mitochondrial dysfunction and ER stress increase ROS production, which enhances NLRP3 expression by up to 2–3 fold in experimental hepatocyte models, triggering caspase-1 activation and secretion of IL-1β and IL-18 [68]. IL-1β, together with Kupffer cell-derived TNF-α, drives necroinflammation and hepatocyte death. Persistent inflammasome signaling correlates with severe DILI phenotypes, contributing to MASH, fibrosis, and cirrhosis [69]. Interest in IL-1 blockade (e.g., anakinra) reflects emerging therapeutic opportunities [70].

### Sphingolipid metabolism disruption

Atorvastatin disrupts sphingolipid metabolism, notably by increasing ceramide by ~ 1.5–2 fold and decreasing S1P, undermining hepatocyte survival [71]. Ceramide promotes apoptosis by activating PP2A and suppressing Akt [72], while reduced S1P weakens cellular defenses. This ceramide–S1P imbalance creates a pro-apoptotic, pro-inflammatory environment, aggravating mitochondrial dysfunction and liver injury [72]. These changes are more pronounced with lipophilic statins than hydrophilic ones, consistent with their hepatic distribution patterns.

### Gut microbiota dysbiosis and enterohepatic cycling

Atorvastatin disrupts gut microbiota and enterohepatic cycling by lowering primary bile acids like cholic acid and CDCA. This favors pro-inflammatory Enterobacteriaceae expansion (up to 3×) and elevates LPS levels [73]. LPS activates TLR4 on Kupffer cells, increasing TNF-α, IL-1β, and IL-6, promoting ROS generation and mitochondrial damage [9, 12]. In parallel, BSEP and NTCP inhibition worsen bile acid imbalance, while reduced secondary bile acids blunt FXR and TGR5 signaling, weakening anti-inflammatory defenses [12]. This gut–liver axis disruption contributes to a chronic inflammatory loop that amplifies injury. Targeting microbiota or bile acid receptors offers a potential therapeutic strategy.

### Epigenetic modifications

Epigenetic changes, including Nrf2 promoter hypermethylation (≈ 50% reduction in expression), diminish antioxidant defense and GSH synthesis [57]. Atorvastatin also downregulates miR-122 and upregulates miR-34a, suppressing SIRT1 and impairing mitochondrial function. These changes increase oxidative stress, inflammation, and senescence [74]. Such epigenetic reprogramming helps explain why some patients develop hepatotoxicity at standard doses, highlighting a role for predictive biomarkers.

### Ferroptosis

Ferroptosis, an iron-dependent cell death process, contributes to atorvastatin-induced injury [14]. Atorvastatin depletes GSH by ~ 40–60% and inhibits GPX4, allowing lipid peroxides to accumulate [75–77]. Iron overload accelerates this via Fenton chemistry. Biomarkers such as PTGS2 and ACSL4 confirm ferroptotic activity [15, 78]. This pathway intersects with mitochondrial ROS signaling, making it a potential therapeutic target in MAFLD and metabolic syndrome contexts.

### Vascular dysfunction and hypoxic injury

Atorvastatin-induced hepatotoxicity also involves vascular dysfunction and hypoxia. Inhibition of the RhoA/ROCK pathway reduces sinusoidal perfusion by ~ 25–30% in experimental models, promoting hypoxia and HIF-1α stabilization [79]. HIF-1α induces VEGF and sinusoidal capillarization, impairing oxygen exchange [80]. Steatosis further reduces diffusion, intensifying oxidative stress and inflammatory injury [81]. This hypoxic contribution complements mitochondrial, bile acid, and inflammatory mechanisms in an integrated hepatotoxicity model.

## Clinical evidence of Atorvastatin-Induced hepatotoxicity

Evaluating clinical data is key to safe atorvastatin use. This section outlines incidence, risk factors, presentation, biomarkers, and management strategies.

### Incidence, risk Factors, and predisposing conditions

Mild ALT/AST elevations (> 3× ULN) occur in 1–3% of users, usually asymptomatic, while severe hepatotoxicity (> 10× ULN) affects approximately 0.1–2%. Liver failure or Hy’s Law cases are rare (< 0.01%) [82]. Risk increases with pharmacokinetic interactions, patient comorbidities, and genetic susceptibility, reflecting a complex interplay between exposure and host factors rather than a purely dose-driven effect.

#### Pharmacokinetic factors

Higher doses correlate with increased AUC, raising hepatotoxicity risk [83]. Atorvastatin’s CYP3A4-dependent metabolism is highly susceptible to interactions with macrolides and azoles, which increase plasma levels and raise DILI risk 3–4× [4]. These data support a dose–exposure–injury relationship, particularly under conditions of impaired clearance.

#### Patient-Specific factors

Pre-existing liver disease such as MAFLD impairs atorvastatin metabolism, heightening risk. Older adults (> 65) have reduced clearance and comorbidities, with ORs of 1.8–2.5 [5]. Metabolic syndrome and diabetes exacerbate inflammation and oxidative stress, amplifying hepatocyte vulnerability. SLCO1B1 CC genotype reduces hepatic uptake, increasing systemic exposure and injury risk (OR ≈ 2.1) [84]. This reflects genotype–phenotype interaction, shifting some hepatotoxicity toward an idiosyncratic rather than purely dose-dependent pattern.

#### Concomitant hepatotoxic drugs

Co-exposure to hepatotoxins such as alcohol or high-dose acetaminophen compounds oxidative stress, increasing injury risk [65]. Patients with these risk factors require intensified monitoring and lower threshold for dose adjustments [85]. Collectively, dose, CYP3A4 inhibition, genetic polymorphisms, and comedications define the individual hepatotoxicity profile.

### Comparison of hepatotoxicity risk across Statins

Structural and pharmacokinetic differences explain variation in statin hepatotoxicity. Atorvastatin, moderately lipophilic and CYP3A4-dependent, shows 1–3% ALT elevation, with risk amplified by DDIs [4]. Simvastatin, also CYP3A4-dependent and more lipophilic, exhibits higher hepatotoxicity (2–5%) and greater DILI risk, making it less favorable in polypharmacy or liver disease [86].

In contrast, rosuvastatin, with minimal CYP450 metabolism and greater hydrophilicity, has a lower risk (< 1%) and better safety in impaired hepatic function [87]. Fluvastatin, metabolized via CYP2C9, shows low to moderate risk but may induce cholestasis [88]. Pravastatin, sulfated rather than CYP-metabolized, has the lowest hepatotoxicity (~ 0.5%) and minimal DDIs, making it preferred in elderly or cirrhotic patients [89].

The higher hepatotoxicity risk of atorvastatin and simvastatin is linked to CYP3A4 metabolism and lipophilicity, whereas rosuvastatin and pravastatin’s favorable profiles are associated with lower hepatic metabolism and reduced passive hepatocellular uptake (Table 4).

**Table 4:** Comparison of hepatotoxicity risk across Statins

Statin	Metabolism	Hepatotoxicity Risk	Impact of CYP3A4 Inhibitors	Suitable for High-Risk Patients
Atorvastatin	CYP3A4	Moderate (1–3% ALT elevation)	Increased risk with CYP3A4 inhibitors (e.g., cyclosporine, azole antifungals, macrolides)	Yes, with liver function monitoring
Simvastatin	CYP3A4	Higher (2–5% ALT elevation)	Increased risk with CYP3A4 inhibitors	Yes, but consider lower doses or alternative statins for high-risk patients
Rosuvastatin	Minimal CYP450 involvement	Low (< 1% ALT elevation)	Minimal impact from CYP3A4 inhibitors	Yes, ideal for chronic liver disease due to low risk
Fluvastatin	CYP2C9	Low to Moderate (higher risk of cholestasis)	Minimal impact from CYP3A4 inhibitors	Yes, for patients at risk of significant DDIs, but monitor for cholestasis
Pravastatin	Sulfation	Lowest (0.5% ALT elevation)	No significant interaction with CYP450 enzymes	Yes, ideal for patients with cirrhosis or liver dysfunction, especially the elderly

ALT: Alanine Aminotransferase, CYP3A4: Cytochrome P450 3A4, CYP2C9: Cytochrome P450 2C9, DDIs: Drug-Drug Interactions

### Clinical presentation and diagnosis: biochemical patterns

Atorvastatin-induced hepatotoxicity ranges from asymptomatic enzyme elevations to severe injury. Elevated ALT and AST—often detected during routine surveillance—are usually the earliest indicators. When symptoms occur, they may include jaundice, fatigue, and right upper quadrant pain [3, 90].

Hepatocellular injury, the most common pattern, is marked by ALT > 3× ULN, often silent but occasionally progressing if persistent. Cholestatic injury, characterized by elevated ALP and bilirubin, typically presents with jaundice and abdominal discomfort. Severe cases (ALT > 10× ULN) are rare but may involve fatigue and jaundice, often reversible upon discontinuation of atorvastatin [91].

Differential diagnosis is essential to rule out viral hepatitis, autoimmune hepatitis, or MAFLD, using serologies, ANA, and imaging [92]. Emerging biomarkers such as miR-122 and HMGB1 may detect injury earlier than conventional enzymes, though they remain investigational [93].

In summary, hepatotoxicity is typically mild and reversible, with a mixed dose-dependent and idiosyncratic pattern. Risk is greatest in genetically or pharmacokinetically susceptible individuals. Accurate diagnosis relies on exclusion of other liver conditions and may soon be enhanced by novel biomarkers such as miR-122 and HMGB1 (Table 5).

**Table 5:** Clinical Presentation, diagnostic Features, and emerging biomarkers of Atorvastatin-Induced hepatotoxicity

Biochemical Pattern	Liver Enzyme Elevation	Symptoms	Severity of Liver Dysfunction	Common Early Indicators	Additional Diagnostic Considerations	Emerging Biomarkers
Hepatocellular Injury (ALT >3x ULN)	ALT >3x ULN, AST may also be elevated	Often asymptomatic, but may present with fatigue and upper abdominal pain	Moderate liver dysfunction, can resolve with drug discontinuation	ALT and AST elevations without symptoms	No specific symptoms, requires routine liver enzyme monitoring for early detection	miR-122 (for hepatocellular injury)
Cholestatic Injury (ALP and Bilirubin Elevation)	Elevated ALP, Bilirubin, with ALT and AST levels less significantly elevated	Jaundice, fatigue, abdominal pain, and more pronounced symptoms	Serious liver dysfunction, potential for long-term damage if untreated	Elevated ALP and bilirubin levels, jaundice	Requires ruling out cholestatic diseases (e.g., MAFLD, viral hepatitis)	miR-122 and HMGB1 for early detection of liver damage
Severe Hepatotoxicity (ALT > 10x ULN)	ALT > 10x ULN, elevated bilirubin, signs of jaundice	Severe jaundice, fatigue, abdominal pain, often with extensive liver damage	Extreme liver dysfunction, liver failure potential, requires immediate discontinuation of statin	Extreme ALT and bilirubin elevations, jaundice, and fatigue	Requires urgent ruling out of viral hepatitis, autoimmune hepatitis, and other liver diseases	miR-122, HMGB1 for detection in early stages

ALT: Alanine Aminotransferase, AST: Aspartate Aminotransferase, ALP: Alkaline Phosphatase, ULN: Upper Limit of Normal, miR-122: MicroRNA-122, HMGB1: High Mobility Group Box 1, ANA: Antinuclear Antibody, MAFLD: Non-Alcoholic Fatty Liver Disease

## Management and monitoring strategies

Managing atorvastatin-induced hepatotoxicity requires balancing the prevention of liver injury with the preservation of its cardiovascular benefits. Key management strategies include dose adjustment, switching to less hepatotoxic statins, pharmacogenomic-informed therapy, and targeted liver function monitoring.

### Dose adjustment and discontinuation criteria

Dose reduction or temporary interruption is advised for mild, asymptomatic ALT elevations (< 3× ULN), whereas discontinuation is indicated when ALT exceeds 3× ULN or if clinical symptoms arise. Severe elevations (ALT > 10× ULN) warrant immediate discontinuation. Enzyme levels typically normalize within weeks following drug withdrawal.

### Switching Statins and adjunctive therapy

In patients who require continued lipid lowering, switching to statins with a lower hepatotoxic potential—such as pravastatin (primarily metabolized by sulfation) or fluvastatin (CYP2C9)—may preserve therapeutic efficacy while minimizing risk. UDCA can be considered in cases of cholestatic injury, although evidence in atorvastatin-induced cholestasis remains preliminary. Simvastatin, which also depends on CYP3A4, carries a similar or slightly higher hepatotoxicity risk, whereas rosuvastatin—with minimal CYP450 metabolism—offers a safer alternative in high-risk patients.

### Pharmacogenomic and clinical monitoring framework

Given atorvastatin’s heavy reliance on CYP3A4 metabolism and OATP1B1-mediated hepatic uptake, pre-treatment pharmacogenomic testing for CYP3A4 and SLCO1B1 variants can help identify patients at increased risk of hepatotoxicity. For example, SLCO1B1 *5/*15 variants are linked to reduced hepatic clearance and higher systemic exposure, which may exacerbate oxidative stress and cholestatic injury. While mild transaminase elevations are typically dose-related, clinically significant hepatotoxicity is largely idiosyncratic and unpredictable. A comprehensive monitoring strategy should begin with baseline assessments, including liver function tests (ALT, AST, ALP, and bilirubin), alongside a review of comedications to detect potential CYP3A4 inhibitors or OATP1B1 competitors that could elevate drug exposure. After initiating therapy or increasing the dose, follow-up testing at approximately 12 weeks is advisable, with additional monitoring targeted toward high-risk individuals such as those with pre-existing liver disease, older age, or genetic risk variants. Drug–drug interactions should be carefully managed, and strong CYP3A4 inhibitors like clarithromycin or itraconazole should be avoided or adjusted, with close clinical follow-up when co-administration is unavoidable. This integrative approach provides a proactive framework for risk stratification, early detection, and individualized management of atorvastatin-related hepatotoxicity.

### Adjunctive hepatoprotective strategies

Antioxidants such as CoQ10, vitamin E, and silymarin have shown promise in reducing oxidative stress, although their clinical utility remains investigational. Their use may be reasonable in research settings or selected high-risk patients pending further evidence.

In summary, An individualized management plan for atorvastatin-induced hepatotoxicity includes dose adjustment or discontinuation, switching to safer statins if necessary, genetic risk stratification, structured liver monitoring, and targeted mitigation of drug interactions. These steps provide a concrete framework for translating mechanistic insights—such as CYP3A4/OATP1B1 involvement—into clinical practice (Table 6).

**Table 6:** Management and monitoring strategies for Atorvastatin-Induced hepatotoxicity

Strategy	Description	When to Consider	Considerations
Discontinuation of Atorvastatin	Stopping atorvastatin typically leads to liver function normalization within weeks. Immediate discontinuation is recommended for ALT > 10x ULN.	For ALT > 10x ULN or persistent liver enzyme elevations, discontinue atorvastatin immediately.	Monitor for symptoms and further liver damage after discontinuation. Investigate alternative causes if enzyme levels don’t normalize.
Dose Adjustment	For mild liver enzyme elevations, reducing atorvastatin dose or temporarily halting therapy can prevent further liver damage.	For mild enzyme elevations and asymptomatic patients, consider dose reduction or temporary pause until liver enzymes normalize.	Assess patient’s response to dose adjustments and consider temporary discontinuation if necessary.
Switching to Alternative Statins (e.g., Pravastatin, Fluvastatin)	Pravastatin and fluvastatin have a lower hepatotoxicity risk and may be safer for patients with liver dysfunction. Close monitoring is required when switching.	For patients unable to tolerate atorvastatin due to liver toxicity, consider switching to pravastatin or fluvastatin, particularly for high-risk populations.	Monitor liver enzymes during the transition and assess patient’s tolerance to the new statin.
Use of Ursodeoxycholic Acid (UDCA)	UDCA may help improve bile flow and reduce bile acid accumulation in the liver, especially in cholestatic injury, but its use is investigational for statin-induced cholestasis.	For cholestatic injury or persistent jaundice, UDCA may be considered, though it remains under investigation.	Carefully evaluate risk vs. benefit when considering UDCA in cholestatic injury and monitor closely.
Monitoring Liver Function Tests (LFTs)	Routine LFT monitoring is essential, particularly for high-risk populations, and can guide decisions on dose adjustments or drug discontinuation.	LFTs should be monitored regularly for patients on high doses, those with pre-existing liver conditions, or those on drugs interacting with statins.	Monitor LFTs regularly during treatment, particularly if symptoms suggest hepatotoxicity. Frequency may increase if liver damage is suspected.
Emerging Hepatoprotective Strategies (e.g., CoQ10, Antioxidants)	CoQ10 and other antioxidants like vitamin E and silymarin may reduce oxidative stress and liver damage, but further research is required to confirm their efficacy.	Consider emerging hepatoprotective strategies for patients with ongoing liver damage or those at high risk, but they should not replace standard care.	Ensure further research and evidence before recommending antioxidants or CoQ10 as a replacement for traditional treatments.

ALT: Alanine Aminotransferase, AST: Aspartate Aminotransferase, LFTs: Liver Function Tests, UDCA: Ursodeoxycholic Acid, CoQ10: Coenzyme Q10

## Research gaps and future prospects

### Personalized medicine for Statin therapy

Genetic polymorphisms in CYP3A4/5 and SLCO1B1, which play key roles in atorvastatin metabolism and hepatic uptake, significantly influence interindividual susceptibility to hepatotoxicity. Integrating pre-treatment pharmacogenomic screening can help identify patients at risk before therapy initiation, enabling dose optimization or early statin switching to minimize hepatic injury. For instance, SLCO1B1**5/15 variants are associated with reduced hepatic clearance and higher systemic exposure, amplifying oxidative stress and mitochondrial vulnerability. Such precision-medicine strategies are particularly valuable in high-risk populations (e.g., elderly, liver disease, polypharmacy) and could enhance both safety and adherence. Importantly, distinguishing patients prone to idiosyncratic versus dose-dependent hepatotoxicity through genetic and phenotypic profiling remains a critical research frontier.

### Mechanisms of hepatotoxicity and drug interactions

Atorvastatin may cause mitochondrial dysfunction, oxidative stress, and disruption of bile acid transporters (e.g., BSEP, NTCP, MRP2), contributing to cholestasis and fibrosis. Targeted therapies addressing these pathways warrant further study. Mechanistic studies directly comparing atorvastatin with other statins could help clarify whether its hepatotoxic profile is unique or part of a broader class effect.

### Impact of gut microbiota and enterohepatic circulation

Atorvastatin may disrupt gut microbiota and enterohepatic circulation, potentially exacerbating liver injury through inflammatory mechanisms. Altered bile acid recycling and liver inflammation are possible consequences. Emerging interventions—such as probiotics or FXR agonists—may offer novel strategies to mitigate statin-induced hepatotoxicity.

### Epigenetic modifications and liver injury

Epigenetic changes—especially dysregulated miRNAs like miR-122 and miR-34a—are linked to hepatocyte apoptosis and oxidative stress. Exploring miRNA-based therapies may lead to novel interventions.

### Long-Term effects of Statins on liver function

While short-term hepatotoxicity is better understood, long-term risks—especially in MAFLD and MASH—remain unclear. Comparative longitudinal studies across different statins are needed to define risk differentials, cumulative injury patterns, and recovery trajectories.

### Translational models and monitoring protocols

Current animal models inadequately reflect human atorvastatin-induced hepatotoxicity, limiting translational relevance. Developing more refined in vitro and in vivo models that incorporate CYP3A4 metabolism, SLCO1B1-mediated transport, and oxidative stress pathways is essential to elucidate mechanisms and test targeted interventions. Equally important is refining clinical monitoring protocols to incorporate baseline pharmacogenomic risk stratification and scheduled LFT monitoring at defined intervals (e.g., baseline, 12 weeks, and targeted follow-up in high-risk groups). Future research should also explore standardized, statin-specific algorithms to distinguish adaptive enzyme elevation from clinically significant injury. Such integration of mechanistic understanding and clinical practice could standardize detection, prevention, and management of atorvastatin-specific liver injury.

### Systems toxicology and omics approaches

Integrating systems toxicology with multi-omics technologies provides a comprehensive framework for understanding atorvastatin-induced hepatotoxicity. Genomic, transcriptomic, proteomic, and metabolomic analyses enable high-throughput identification of molecular disturbances underlying oxidative stress, inflammation, and mitochondrial dysfunction.

Despite progress, multi-omics applications in atorvastatin hepatotoxicity remain limited. Large-scale studies combining omics data with clinical phenotypes are needed to clarify mechanisms and identify predictive biomarkers. Proteomic profiling may reveal early injury signatures, while transcriptomic and metabolomic analyses define stress-response and metabolic alterations. Genomic studies also highlight susceptibility variants linked to hepatic risk.

Integrative systems biology models that merge molecular, genetic, and environmental factors will improve prediction of hepatotoxicity and support individualized risk assessment. Advancing these frameworks toward clinical translation is a key goal in precision toxicology.

## Study limitations

Most studies emphasize short- to medium-term effects, leaving long-term liver outcomes—especially in conditions like MAFLD and MASH—underexplored. Population heterogeneity in genetics, comorbidities, and ethnicity limits generalizability, highlighting the need for diverse, targeted research. Although polymorphisms in SLCO1B1, CYP3A4, and UGT1A1 are linked to risk, their interaction with environmental and lifestyle factors remains unclear. The absence of standardized liver monitoring protocols adds variability to clinical practice. Promising adjunct therapies like antioxidants require further validation to confirm long-term safety and efficacy.

### Summary

Atorvastatin effectively reduces cardiovascular events but can cause liver enzyme elevations and, rarely, severe hepatotoxicity. These effects are usually mild and reversible, though rare cases of liver failure highlight the need for vigilance. Hepatotoxicity arises from complex mechanisms, including oxidative stress, mitochondrial damage, and inflammatory responses, with genetic and drug interaction factors modulating individual risk. While clinically significant liver injury is rare, early enzyme elevations are relatively common and may prompt unnecessary discontinuation. Balancing statin benefits with hepatic safety involves personalized risk assessment, especially for patients with pre-existing liver disease or polypharmacy. Future directions should focus on genetic screening, biomarker development, and targeted hepatoprotective strategies, enabling safer long-term use while maintaining atorvastatin’s proven cardiovascular efficacy.

## Conclusion

Atorvastatin remains a pivotal therapy in the management of hyperlipidemia and cardiovascular disease due to its proven clinical efficacy. However, despite its favorable safety profile, hepatotoxicity—though infrequent—poses clinically relevant risks. Evidence indicates that atorvastatin-induced liver injury results from a multifactorial interplay involving mitochondrial dysfunction, oxidative stress, bile acid transporter disruption, and inter-individual genetic variability. To mitigate these risks, a personalized approach integrating pharmacogenomic screening, risk-adapted monitoring, and individualized dosing is essential. Such strategies not only enhance patient safety but also preserve therapeutic outcomes. The cardiovascular benefits of atorvastatin continue to outweigh the hepatic risks in the majority of patients; however, greater focus on predictive biomarkers, molecular risk profiling, and targeted hepatoprotective adjuncts is warranted. Advancing these precision strategies will support safer statin therapy and further the integration of mechanistic toxicology into clinical practice.

## Data Availability

No datasets were generated or analysed during the current study.

## Abbreviations

ACSL4: Acyl-CoA Synthetase Long Chain Family Member 4
ASK1: Apoptosis Signal-Regulating Kinase 1
Bak: Bcl-2 Antagonist Killer
Bax: Bcl-2-Associated X Protein
Bcl-2: B-cell lymphoma 2
BSEP: Bile Salt Export Pump
Caspase-1: Cysteinyl Aspartate Specific Proteinase 1
CoQ10: Coenzyme Q10
CVDs: Cardiovascular Diseases
CYP3A4: Cytochrome P450 3A4
FXR: Farnesoid X Receptor
GSH: Reduced glutathione
HIF-1α: Hypoxia-Inducible Factor 1 Alpha
HMG-CoA: 3-Hydroxy-3-Methyl-Glutaryl-Coenzyme A
JNK: c-Jun N-terminal Kinase
LDL: Low-Density Lipoprotein
LPS: Lipopolysaccharides
MAPK: Mitogen-Activated Protein Kinase
MRP: Multidrug Resistance-Associated Protein
MAFLD: Metabolic Dysfunction–Associated Fatty Liver Disease
MASH: Metabolic Dysfunction–Associated Steatohepatitis
NF-κB: Nuclear Factor Kappa-Light-Chain-Enhancer of Activated B Cells
NLRP3: NOD-Like Receptor Family Pyrin Domain Containing 3
NTCP: Sodium-Taurocholate Cotransporting Polypeptide
OATP1B1: Organic Anion Transporting Polypeptide 1B1
PARP: Poly (ADP-ribose) Polymerase
SIRT1: Sirtuin 1
SLCO1B1: Solute Carrier Organic Anion Transporter Family Member 1B1
SOD2: Superoxide Dismutase 2
SULT2A1: Sulfotransferase Family 2 A Member 1
TGF-β: Transforming Growth Factor Beta
TLR4: Toll-Like Receptor 4
UDPGT1A1: UDP-Glucuronosyltransferase 1A1
VEGF: Vascular Endothelial Growth Factor
